# Quantification of Age and Sex Ratio Differences between Trial and Target Population for New Drugs

**DOI:** 10.1002/cpt.70221

**Published:** 2026-02-01

**Authors:** Miquel Serra‐Burriel, Paul Schlossmacher, Kerstin Noelle Vokinger

**Affiliations:** ^1^ Epidemiology, Biostatistics, and Prevention Institute University of Zurich Zurich Switzerland; ^2^ Academic Chair for Regulation in Law, Medicine and Technology University of Zurich, Faculty of Law Zurich Switzerland; ^3^ Academic Chair for Regulation in Law, Medicine and Technology, Department of Health Sciences and Technology, ETH Zurich Zurich Switzerland

## Abstract

Clinical trials are essential to understand the benefit‐harm profile of new drugs. Lack of adequate representation in age and sex ratio in clinical trials can result, for example, in higher side effects for underrepresented patients. We quantified differences in age and sex ratios between trial and target populations of new drugs approved by the FDA 2011–2022. We used the FDA's database to identify all new drugs and pivotal randomized trials. Information for average age and sex ratio was obtained from clinicaltrials.gov. Each trial's indication was matched with prevalence estimates of the targeted diseases from the global burden of disease study. A total of 458 drugs (773 trials) were included. The trial populations were significantly younger, on average 4.8 years (95% CI [5.4 years, 4.2 years]), and the female ratio significantly smaller, on average 4.3 percentage points (95% CI [5.4 pp, 3.3 pp]), than the target populations. For diseases with average patient age below 40, the trial population was significantly older than the target population but significantly younger 40 years and older. For diseases with average age between 30 and 39, the female ratio in the trial population was significantly higher than in the target population but significantly lower 50 and older. Better age and sex ratio representation in the trial population is indicated to improve safety and efficacy for patients. Trials targeting diseases below 40 should enroll younger participants and increase their male ratio, while the opposite is true for trials targeting diseases with an older age.

**Study Highlights**

**WHAT IS THE CURRENT KNOWLEDGE ON THE TOPIC?**

Clinical trials are essential to understand the benefit‐harm profile of new drugs. Lack of adequate representation in age and sex ratio in clinical trials can result, for example, in lower efficacy or higher side effects for underrepresented patients. Previous studies found that, for a selected number of diseases, older patients and female participants are often underrepresented in clinical trials.

**WHAT QUESTION DID THIS STUDY ADDRESS?**

We aimed to quantify the differences in age and sex ratio between trial and target populations for new drugs approved by the FDA between 2011 and 2022, and analyzed their differences depending on the average patient age of diseases.

**WHAT DOES THIS STUDY ADD TO OUR KNOWLEDGE?**

Better age and sex ratio representation in the trial population is indicated to improve safety and efficacy for patients in the target population. Trials targeting diseases below 40 should enroll younger participants and increase their male ratio, while the opposite is true for trials targeting diseases with an older age.

**HOW MIGHT THIS CHANGE CLINICAL PHARMACOLOGY OR TRANSLATIONAL SCIENCE?**

The study findings could provide guidance for the improvement of the design of clinical studies with the ultimate objective of improving health outcomes for patients irrespective of their age and sex.

Clinical trials are essential to understand the benefit‐harm profile of new drugs.^1^, ^2^, ^3^ Ideally, the population in a clinical trial represents the target population to ensure the validity and applicability of the trial results in the clinical setting.^4^, ^5^, ^6^ Due to sex‐specific differences in disease patterns, metabolism, and drug pharmacokinetics, female patients may experience unanticipated adverse events and lower survival based on study results with overrepresentation of male participants.^7^, ^8^, ^9^ It is also relevant that the age of participants enrolled in the clinical trials is representative of the target population. Drug efficacy can be lower and the risk of side effects higher in the target population if younger participants are disproportionately enrolled compared to the average age of patients affected by the disease.^4^, ^10^, ^11^, ^12^

Previous studies found that, for a selected number of diseases, older patients and female participants are often underrepresented in clinical trials.^10^, ^12^, ^13^, ^14^, ^15^ However, age and sex ratio differences between trial and target populations have not been comprehensively quantified across therapeutic areas. It also remains unclear whether diseases with a younger average patient age have larger discrepancies in age and sex ratio representation compared to the trial population than diseases with older average patient age. This information is important to identify which clinical trials should be prioritized to improve age and sex ratio representation.

In this study, we aimed to quantify the differences in age and sex ratio between trial and target populations for new drugs approved by the FDA between 2011 and 2022. We also categorized diseases in groups based on their average patient age to analyze whether there were differences in age and sex ratio between trial and target populations depending on the average patient age of diseases.

## MATERIALS AND METHODS

### Data

We used the FDA's public database to identify all new drugs that were approved between January 2011 and December 2022.^16^ All phase III and randomized phase II trials relevant for the drugs' approval were extracted from the FDA's public database.^17^

Information for average age and sex ratio of trial participants was obtained from clinicaltrials.gov.^18^ We manually matched each trial's indication with prevalence estimates of the targeted diseases for the United States from the Global Burden of Disease (GBD) 2021 study.^19^ Prevalence data from GBD was matched using the year of the start date of the trial, that is, if a trial started in 2019, age and sex ratio information was matched with prevalence data from 2019 for the targeted disease from the GBD study.

GBD provides age‐specific prevalence estimates in 5‐year intervals (0–4 years, 5–9 years, 10–14 years, etc.). Using the midpoint of each interval, we estimated the average age for each condition by computing a weighted mean across these midpoints, using each age group's population as weights. We also estimated the female ratio for the target population using the prevalence for the sex groups “male” and “female” from the GBD data. All analyses were based on the US target population. To assess the robustness of our estimates, we repeated the analyses using prevalence data for Germany and Japan, which, after the United States, are among the countries with the highest amount of clinical trial sites.

To avoid bias from trials with extreme age eligibility, we included only trials with a minimum age requirement between 18 and 65 years. To align the target population with this inclusion criteria, we recalculated the average age using only GBD age bands older or equal than 15 years. We opted to include the 15–19 age group instead of the 20–24 as a conservative data processing decision, estimating a target population slightly younger.

Trials were categorized in therapeutic areas based on the World Health Organization's Anatomical Therapeutic Chemical (ATC) classification system to assess differences between cancer and non‐cancer diseases.^20^ We differentiated between these two groups because cancer drugs have been the largest therapeutic group receiving approval in recent years.^21^, ^22^, ^23^

### Statistical analysis

Descriptive statistical analysis was conducted to quantify the differences between trial and target population for age and sex ratio. They were calculated as average age of the trial population minus average age of the target population, and female‐to‐male ratio in the trial population minus female‐to‐male ratio in the target population. The analyses were conducted for all targeted diseases as well as for cancer vs. non‐cancer diseases. If a trial targeted more than one disease, an unweighted average of the difference for each targeted disease was calculated.

To assess whether the differences in average age between trial and target population varied across average age groups of diseases, we categorized the targeted diseases by average age groups (30–39, 40–49, 50–59, 60–69, 70+). If a trial targeted more than one disease, we took the average age for each disease and calculated an unweighted average age over all conditions to find the correct age‐group bracket for the trial.

We computed 95% confidence intervals for the true differences via the nonparametric bootstrap with 1,000 draws as implemented in the “basic” method of the “boot” package in R.^24^, ^25^ Differences were considered statistically significant when the confidence interval did not include 0, based on the duality between confidence intervals and hypothesis testing.

Statistical analyses were performed in R, version 4.4.0 (R Foundation for Statistical Computing). The study was exempt from institutional review board approval because it used nonidentifiable data and did not constitute human participants research.

## RESULTS

### Overview

**Figure 1:**
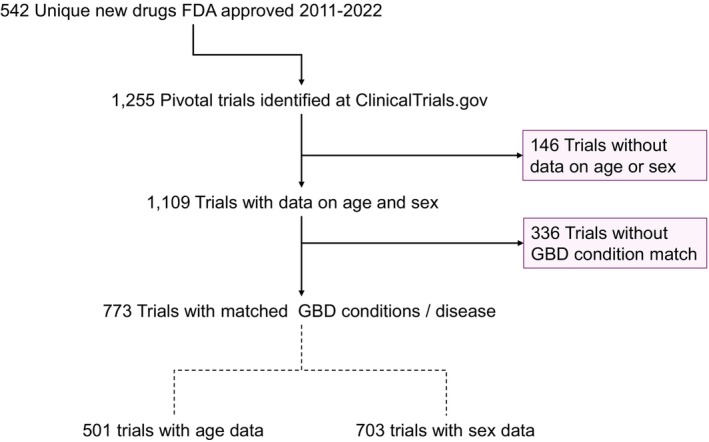
Trial selection flowchart. GBD, global burden of disease; FDA, US Food and drug administration.

We identified 542 new drugs that were approved between 2011 and 2022, which corresponded to 1,255 phase III and randomized phase II trials. Of the 1,255 trials, 1,109 had information on clinicaltrials.gov about mean age and sex ratio. We were able to match 773 of 1,109 trials with the targeted conditions from the GBD study. A total of 773 trials (458 drugs; 501 trials for age and 703 trials for sex ratio) were included in our study cohort (**Figure** **1**). A total of 207 trials (136 drugs) were indicated for treatment of a cancer condition. The number of trials for age and sex ratio were different because not all trials provide information for both characteristics. The main characteristics of the included trials are described in **Table** **1**.

**Table 1:** Main characteristics of included trials

	*n*	(%)
Trials	773	
Phase 3	554	71.7%
Industry sponsor	759	98.2%
Patient enrolment (median [IQR])	453	[221; 795]
Year of trial start		
2006	30	3.9%
2007	47	6.1%
2008	70	9.1%
2009	77	10.0%
2010	78	10.1%
2011	52	6.7%
2012	45	5.8%
2013	61	7.9%
2014	60	7.8%
2015	60	7.8%
2016	54	7.0%
2017	39	5.0%
2018	40	5.2%
2019	32	4.1%
2020	12	1.6%
Number of target conditions		
1	582	75.3%
>1	191	24.7%
Oncological trial	207	26.8%
Top 10 conditions		
Type 2 diabetes	91	11.8%
COPD	24	3.1%
Rheumatoid arthritis	23	3.0%
Psoriasis	18	2.3%
Breast cancer	17	2.2%
Multiple myeloma	14	1.8%
Epilepsy	13	1.7%
Asthma	11	1.4%
Migraine	11	1.4%
Ocular hypertension	10	1.3%

COPD, chronic obstructive pulmonary disease; IQR, interquartile range.

### Quantification of age differences between trial and target population

The trial population was significantly younger, on average 4.8 years (95% CI [5.4 years, 4.2 years]), than the target population. No notable difference was observed between cancer and non‐cancer trials (**Table** **2**).

**Table 2:** Overview of age and sex ratio differences between trial and target population

Age			
	*n* trials	Δ – Age	95%‐CI
Overall	501	−4.8 years	[−5.4 years, −4.2 years]
Cancer	85	−4.7 years	[−6.0 years, −3.3 years]
Non‐cancer	415	−4.9 years	[−5.5 years, −4.2 years]

Sex ratio			
	*n* trials	Δ – Female ratio (pp)	95%‐CI
Overall	703	−4.3 pp	[−5.4 pp, −3.3 pp]
Cancer	156	+0.6 pp	[−1.4 pp, +2.5 pp]
Non‐cancer	547	−5.7 pp	[−7.0 pp, −4.6 pp]

Negative numbers mean that the trial age was younger or had a lower female ratio in the trial population compared to the target population.

CI, confidence interval; pp, percentage points.

**Figure 2:**
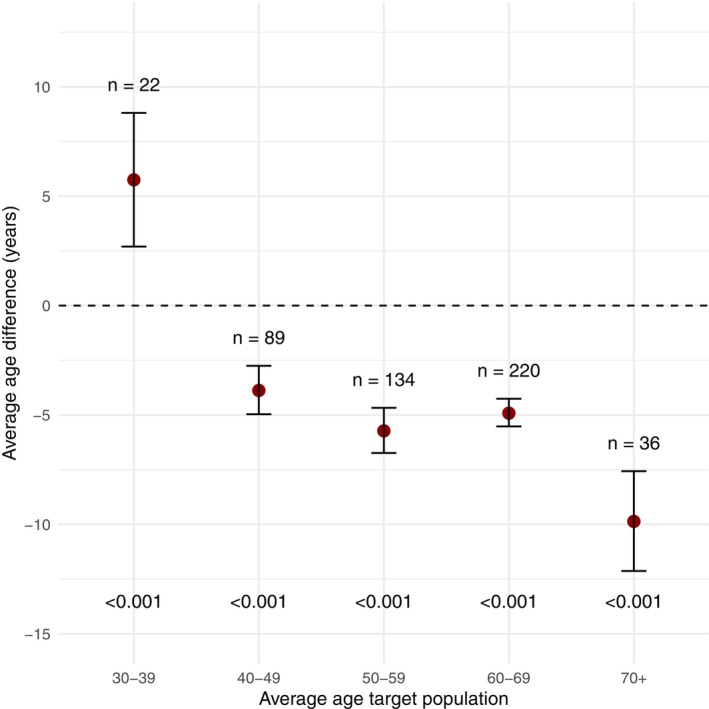
Differences in age between trial and target population. Plot represents the point estimates and corresponding confidence intervals of differences in years between the trial and target population. Differences above 0 mean that the trial population was older than the target population. Differences below 0 mean that the trial population was younger than the target population.

When categorizing the diseases based on their average patient age, the trial population was 5.8 years older than the target population for diseases with an average patient age between 30 and 39, 3.9 years younger for diseases with an average patient age between 40 and 49, 5.7 years younger for diseases with an average patient age between 50 and 59 years, 4.9 years younger for diseases with an average patient age between 60 and 69 years, and 9.9 years younger for diseases with an average patient age of 70+ years. These age differences between trial and target population were statistically significant at a 5% level for all age categories (**Figure** **2**).

When weighting the trials by their sample size, the trial population was on average 3.8 years (95% CI [4.9 years, 2.6 years]) younger than the target population.

The age difference between trial and target population was similar for cancer and non‐cancer trials (**Table** **2**).

### Quantification of sex ratio differences between trial and target population

The female ratio in the trial population was on average 4.3 percentage points (95% CI [5.4 pp, 3.3 pp]) smaller than in the target population (**Table** **2**). The percentage points reflect the difference between the female ratio in the trial population vs. the female ratio in the target population. For example, the trial NCT02512510, a drug (revefenacin) indicated for treatment of chronic obstructive pulmonary disease, had a female ratio of 50.5%, while in 2015 (year of trial start) the overall population with this disease had a female ratio of 54.8%. This results in a difference of 4.3 percentage points.

**Figure 3:**
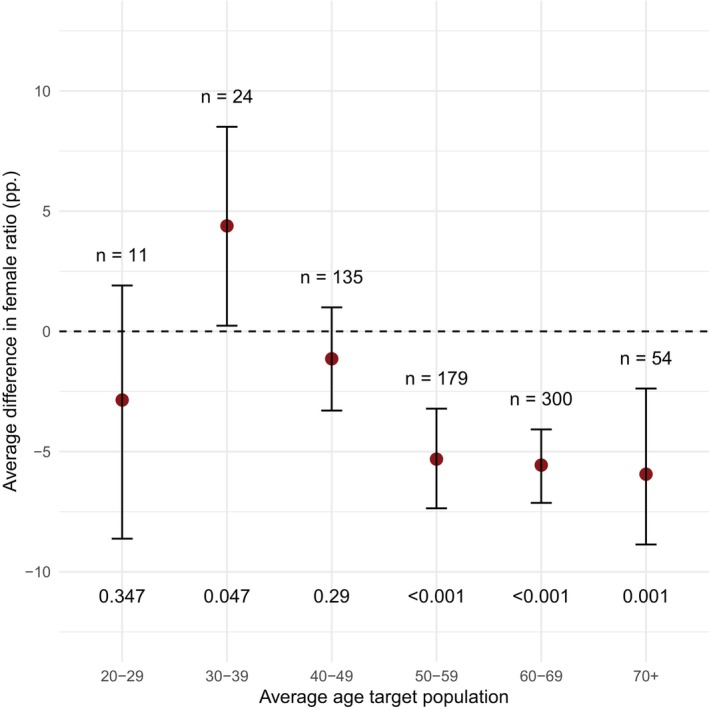
Differences in sex ratio between trial and target population. Plot represents the point estimates and corresponding confidence intervals of differences in years between the trial and target population. Percentage points above 0 mean that the female ratio was higher in the trial population compared to the target population. Percentage points below 0 mean that the female ratio was lower in the trial population compared to the target population.

When categorizing the diseases based on their average patient age, the female ratio in the trial population was 2.9 percentage points lower than in the target population for diseases with an average patient age between 20 and 29 years, and 4.4 percentage points higher for diseases with an average patient age between 30 and 39 years. Female ratio in the trial population was 1.1 percentage points lower than in the target population for diseases with an average patient age between 40 and 49 years and further decreased for diseases with higher average patient age (5.3 percentage points for 50–59, 5.6 percentage points for 60–69, and 6 percentage points for 70+). These sex differences between trial and target population were statistically significant at the 5% level for all age categories with the exception of 20–29 and 40–49 years (**Figure** **3**).

When weighting the trials by their sample size, the female ratio in the trial population was on average 8.2 percentage points smaller than in the target population (95% CI [10.8 pp, 5 pp]).

The difference in sex ratio was different for cancer and non‐cancer trials. For cancer trials, the female ratio in the trial population was on average 0.6 percentage points higher than in the target population, a nonsignificant difference at the 5% level between the trial and target population. For non‐cancer trials, the female ratio in the trial population was on average 5.7 percentage points smaller than in the target population, a significant difference at the 5% level between trial and target population (**Table** **2**).

### Sensitivity analysis

When conducting the same analyses for the target population in Germany, the trial population was on average 6.7 years (95% CI [7.3 years, 6.1 years]) younger in the trial population than in the target population, and in Japan the trial population was on average 8.9 years (95% CI [9.4 years, 8.2 years]) younger than in the target population.

The results were also similar when conducting the sensitivity analysis for sex ratio. The trial population had 3.8 percentage points fewer females (95% CI [4.8 pp, 2.7 pp]) than the target population in Germany. With the target population in Japan, the difference was −2.4 percentage points (95% CI [−3.5 pp, −1.4 pp]).

## DISCUSSION

The study estimates showed that the trial population was on average approximately 5 years younger than the target population. However, differences were observed depending on the average age of the targeted disease. For diseases with an average patient age below 40 years, the trial population was significantly older than the target population. By contrast, for diseases with higher average patient age, the trial population was significantly younger than the target with the largest age difference (approximately 10 years) for diseases with an average patient age of 70+ years. The female sex ratio was on average 4 percentage points smaller than in the target population, with differences between the average patient age of targeted diseases. For diseases with an average age between 30 and 39, the female ratio in the trial population was significantly higher than in the target population, while it was significantly lower for diseases with average patient age of 50 years and older. Our findings suggest that age and sex ratio representation between trial and target population could be improved.

We found differences depending on the average age of the disease; thus, generalizing across all diseases may blur the actual differences between both population groups. While trials targeting diseases with an average age below 40 should improve their population representation by enrolling younger participants and, if indicated, increasing the male ratio, trials targeting diseases with an average age above 40 could improve their population representation by increasing the female ratio and enrolling older participants.

An example for a drug from our study cohort intended for treatment of a disease for patients with an average age of 70+ is aflibercept for treatment of age‐related macular degeneration. Its clinical trial (NCT00637377) had an average age of 73.9 years, while the target population had an average age of 78.8 years. Thus, the average age in the patient population was 4.9 years lower than in the target population. A potential explanation why the trial population was significantly younger than the target population for diseases with an older average patient age could be the exclusion criteria in clinical trials, which often encompass multimorbidity, concomitant medication use, and polypharmacy.^11^, ^26^ Especially, multimorbidity as well as other exclusion criteria increase with age.^11^ Such stringent exclusion criteria in clinical trials do not appropriately account for the heterogeneity of vulnerable characteristics observed in the target population. Rather, a better representation in the trial population is indicated to increase the completeness of evidence‐based drug assessments to evaluate safety and efficacy for patients in the target population.

In our study, more than a third of the trials (8/22) targeting diseases with an average age between 30 and 39 years were indicated for treatment of major depressive disorder. Studies showed that men tend to avoid recognizing their illness, in part because traditional depressive symptoms (e.g., sadness) are at odds with societal ideals of masculinity.^27^, ^28^, ^29^, ^30^ These factors could explain why fewer men participated in the trial population compared to the prevalence of male patients in the target population. Our findings are also aligned with a previous study, which showed that men are substantially underrepresented in clinical trials targeting depression.^31^ Thus, moving forward it is important to increase the number of male participants in trials targeting depression to assess safety and efficacy of the drugs adequately for the target population. By contrast, for trials targeting diseases with a higher average age, more female participants should be included in clinical trials. For example, the female ratio for the clinical trial NCT01539512 (idelalisib) was 34.5%, while the female ratio for the target population was 40.3%. Idelalisib is indicated for treatment of chronic lymphocytic leukemia, a disease with an average age 70 +.

Prior studies emphasized the importance that the population in clinical trials represents the target population with regard to sex ratio and age to ensure the validity and applicability of the trial results in the clinical setting.^4^, ^5^, ^6^ Research found, for example, that female patients who were administered anti‐tumor necrosis factor for treatment of inflammatory bowel disease might be more at risk for allergic adverse drug events compared to male patients.^32^, ^33^ Another example is zolpidem, which is indicated to treat insomnia. Sex differences in the metabolism of zolpidem result in slower drug clearance and greater next day impairment in female patients compared to male patients.^34^ Previous studies also showed the relevance of adequate age representation in clinical trials.^4^, ^35^, ^36^ One study that analyzed 186,339 clinical trials demonstrated that the adverse event diversity increased at an average rate of 77% for each age group (30–39 years, 40–49 years, 50–59 years, 60–69 years).^36^

## LIMITATIONS

We manually matched the indications of the trials with prevalence estimates of the targeted diseases for the United States from GBD. GBD includes 337 different causes or conditions. Thus, it is possible that the GBD condition is broader than the indication of the clinical trial. For example, “non‐small lung cancer” (clinical trial) was matched with “tracheal, bronchus, and lung cancer” (GBD). However, we did not expect the matching of specific diseases with broader categories to introduce specific biases in the direction of age or sex ratio because the limitations of matching were not focused on specific diseases but rather a general limitation. Furthermore, if a trial targeted more than one disease, it remained unclear how many patients in the trial were affected by each disease. In such cases, we calculated the difference in age and female ratio for each of those conditions and then reported an unweighted average over those differences. A similar limitation occurred when we assigned trials to age groups in our analyses by age group. Whenever a trial had multiple conditions, we calculated an unweighted average age over all conditions. We limited our analysis to age and sex. We did not account for other important characteristics, for example, race. Finally, because sex and gender data were not disaggregated by country in multinational trials, country‐specific estimates should be interpreted with caution.

## CONCLUSION

The study findings showed differences in age and sex ratio between trial and target population depending on the average patient age of the targeted disease. For diseases with an average patient age below 40 years, the trial population was significantly older than the target population, while the trial population was significantly younger than the target population for diseases with higher average patient age. For diseases with an average age between 30 and 39, the female ratio in the trial population was significantly higher than in the target population, while it was significantly lower for diseases with an average patient age of 50 years and older. These findings indicate that a nuanced assessment is necessary when designing and conducting a clinical trial and deciding on the age and sex ratio representation with the objective to ensure the validity and applicability in the clinical setting.

## CONFLICT OF INTEREST

The authors declared no competing interests for this work.

## FUNDING

This study was partially funded by the Swiss National Science Foundation (SNSF, grant number PCEGP1_194607).

## AUTHOR CONTRIBUTIONS

M.S.B. and K.N.V. designed the research; M.S.B. and P.S. performed the research; M.S.B. and P.S. analyzed the data; K.N.V., M.S.B., and P.S. wrote the manuscript.
